# Comprehensive analysis of m^6^A circRNAs identified in colorectal cancer by MeRIP sequencing

**DOI:** 10.3389/fonc.2022.927810

**Published:** 2022-08-22

**Authors:** Feng He, Qin Guo, Guo-xiu Jiang, Yan Zhou

**Affiliations:** ^1^ The First Affiliated Hospital of Chengdu Medical College, School of Clinical Medicine, Chengdu Medical College, Chengdu, China; ^2^ National Health Commission (NHC), Key Laboratory of Nuclear Technology Medical Transformation, Mianyang Central Hospital, School of Medicine, University of Electronic Science and Technology of China, Mianyang, China

**Keywords:** colorectal cancer, MeRIP sequencing, M^6^A modification, CircRNA, RNA-Seq

## Abstract

**Purpose:**

To characterize the entire profile of m^6^A modifications and differential expression patterns for circRNAs in colorectal cancer (CRC).

**Methods:**

First, High-throughput MeRIP-sequencing and RNA-sequencing was used to determine the difference in m^6^A methylome and expression of circRNA between CRC tissues and tumor-adjacent normal control (NC) tissues. Then, GO and KEGG analysis detected pathways involved in differentially methylated and differentially expressed circRNAs (DEGs). The correlations between m^6^A status and expression level were calculated using a Pearson correlation analysis. Next, the networks of circRNA-miRNA-mRNA were visualized using the Target Scan and miRanda software. Finally, We describe the relationship of distance between the m^6^A peak and internal ribosome entry site (IRES) and protein coding potential of circRNAs.

**Results:**

A total of 4340 m^6^A peaks of circRNAs in CRC tissue and 3216 m^6^A peaks of circRNAs in NC tissues were detected. A total of 2561 m^6^A circRNAs in CRC tissues and 2129 m^6^A circRNAs in NC tissues were detected. Pathway analysis detected that differentially methylated and expressed circRNAs were closely related to cancer. The conjoint analysis of MeRIP-seq and RNA-seq data discovered 30 circRNAs with differentially m^6^A methylated and synchronously differential expression. RT-qPCR showned circRNAs (has_circ_0032821, has_circ_0019079, has_circ_0093688) were upregulated and circRNAs (hsa_circ_0026782, hsa_circ_0108457) were downregulated in CRC. In the ceRNA network, the 10 hyper-up circRNAs were shown to be associated with 19 miRNAs and regulate 16 mRNAs, 14 hypo-down circRNAs were associated with 30 miRNAs and regulated 27 mRNAs. There was no significant correlation between the level of m^6^A and the expression of circRNAs. The distance between the m^6^A peak and IRES was not significantly related to the protein coding potential of circRNAs.

**Conclusion:**

Our study found that there were significant differences in the m^6^A methylation patterns of circRNAs between CRC and NC tissues. M^6^A methylation may affect circRNA-miRNA-mRNA co-expression in CRC and further affect the regulation of cancer-related target genes.

## Introduction

The latest epidemiological data in 2020 show that colorectal cancer (CRC) is the third most common cancer in the world, with more than 1.93 million new cases, accounting for 9.7% of the world’s newly diagnosed cancers (1). Treatment of CRC includes surgery, radiation therapy, and chemotherapy (2). However, approximately one-quarter of patients have developed liver metastases at the time of first diagnosis, and patients with advanced CRC have a poor 5-year survival and quality of life (3, 4). This highlights the need for a better understanding of the underlying pathogenic mechanisms that promote the development of CRC to identify and treat CRC in the early stage.

N6-Methyladenosine (m^6^A), methylated adenosine at the N^6^ position, is the most abundant internal modification of RNAs in eukaryotes (5, 6). Methylation is a reversible epigenetic modification that affects the fate of modified RNAs (7). Methylation involes in RNAs behaviors regulation, such as pre-mRNA splicing, polyadenylation, regulation of RNA stability and long noncoding RNAs biological functions (8–10). In recent years, the function of methylation has been extensively studied in the progression of various cancers, including leukemia, glioma, breast cancer and liver cancer (11–14). M^6^A modification plays a double role in human carcinogenesis and suppression. Methyltransferases acting as “writers” and demethylases acting as an “erasers” are important for maintaining balanced Methylation. M^6^A reader proteins specifically recognize m^6^A transcripts and further regulate gene expression and tumor development (15).

Circular RNAs (circRNAs) characterized by a covalently closed loop produced *via* back-splicing are a novel class of non-coding RNAs. The connection between the 5’cap and the 3’end make them more stable and not susceptible to degradation (16, 17). CircRNAs can accumulate at high levels in cells and can also be detected in the sera and exosomes (18). Therefore, circRNAs may be a viable candidate as tumor biomarkers. In the past, circRNAs were considered a byproduct but recent evidence suggest that they play a key role in various biological functions, such as acting as microRNA sponges (19, 20), transcriptional regulators (21–23), and translational intermediates (24–26). Multiple studies have shown that circRNAs disorders are involved in cancer progression and tumor chemotherapy resistance (19–23, 27, 28).

M^6^A modifications are also widespread in both circRNAs and mRNAs. M^6^A modifications are also widely present in circular RNAs and may affect tumorigenesis and development through various mechanisms. Current research shows that activation of YAP1 by N^6^-Methyladenosine-Modified circCPSF6 Drives Malignancy in Hepatocellular Carcinoma (29). CircMETTL3, upregulated in an m^6^A-dependent manner, promotes breast cancer progression. However, the mechanism of cicRna methylation involved in CRC is still in the preliminary exploratory stage (30). Here, we attempted to characterize the patterns of m^6^A modification in circRNAs from CRC tissues and tumor-adjacent normal control tissues (NC). At the same time, we analyze the relationship between m^6^A modification and the expression and encoding potential of circRNAs in CRC. We expect that this study can lay a foundation for exploring the oncogenic mechanism of CRC.

## Materials & methods

### Tissue samples

Five colorectal cancer specimens and adjacent normal tissue were obtained at the Chengdu Medical College from March 2017 to June 2018. None of these patients received radiation or chemotherapy before the specimens were collected. The collected specimens were frozen sectioned and stained with hemAtoxylin-eosin to confirm that the collected specimens were CRC tissues and corresponding normal tissues adjacent to the cancer. This study has been approved by the Ethics Committee of the First Affiliated Hospital of Chengdu Medical College. The clinicopathological data of 5 patients with CRC are the same as the article (doi: 10.3389/fcell.2021.760912) published by our team in Front Cell Dev Biol.

### Extraction of total RNA

RNA was extracted using TRIzol Reagent (Invitrogen, Carlsbad, CA, USA) according to the manufacturer’s instructions. RNA concentration was determined using a NanoDrop ND-1000 spectrometer (Thermo, Waltham, MA, USA), and RNA integrity was evaluated by denaturing gel electrophoresis.

### MeRIP-seq & RNA-seq

High-throughput RNA sequencing was performed by Cloud-Seq Biotech (Shanghai, China). M^6^A RNA immunoprecipitation was performed using the GenSeqTM m^6^A -MeRIP Kit (GenSeq, Beijing, China) according to the manufacturer’s instructions. The input samples without immunoprecipitation and the m^6^A IP samples were used as templates for the NEBNext^®^ Ultra II Directional RNA Library Prep Kit (New England Biolabs, Inc., USA). These libraries were controlled for quality and quantified using the BioAnalyzer 2100 system (Agilent Technologies, Inc., Palo Alto, CA, USA). Then, library sequencing was performed on an Illumina HiSeq instrument with 150bp paired end reads.

### Data analysis

After 3’ adaptor-trimming and the removal of any low-quality reads by the cutadapt software (v1.9.3), the clean reads were aligned to the reference genome using STAR software (v2.5.1b), and the circRNAs were detected and identified using DCC software (v0.4.4). The data were normalized with edgeR(v3.16.5) software and differentially expressed circRNAs with statistical significant expression changes (p ≤ 0.05 and fold change≥2) were identified. MACS (v1.4.2) software was used to identify methylated sites on the RNAs (m^6^A peaks) and differentially methylated sites were identified using diffReps software (v1.55.3) (P ≤ 0.05 and fold change≥2). Gene ontology (GO) and Kyoto Encyclopedia of Genes and Genomes (KEGG) pathway enrichment analysis were performed by DAVID database.

### Validation of circRNAs expression by RT-qPCR

Total RNA was extracted from 8 pairs of CRC and NC tissues using Tatal RNA Extration KIT (Solarbio, Beijing, China) according to the manufacturer’s instructions. The concentration and purity of the RNA was determined using Nanodrop 2000 microspectrophotometer (Thermo Scientific, New York, USA). The reversing transcription was performed according to the PrimeScript RT reagent kit with gDNA Eraser (Takara Biotechnology Co., Ltd, Beijing, China). Real-time quantitative PCR (RT-qPCR) was performed using TB Green™ Premix Ex Taq™ II (Takara Biotechnology Co., Ltd, Beijing, China). All RT-qPCR analyses were conducted in triplicate and the average value was calculated. The expression values of genes were normalized GAPDH and then 2−ΔΔCt transformed for the gene expression.

### M^6^A correlation, ceRNA network, and coding potential prediction

The differences in the m^6^A fold enrichment for the total circRNAs populations between the CRC and NC groups were used to create a correlation map linking M^6^A status and expression level. We selected 10 hypermethylated circRNAs with upregulated expression and 14 hypo-methylated circRNAs with downregulated expression to establish the circRNA-miRNA-mRNA network. The regulatory relationships between the circRNA-miRNA-mRNA were visualized using the Target Scan (v8.0) and miRanda (v3.3a) software (31). Then, The correlations between m^6^A status and expression level in each of the groups were calculated using a Pearson correlation analysis. we used LGC software to predict the coding potential of circular RNA (32).

## Results

### General characteristics of circRNA m^6^A modification patterns in CRC and NC tissues

**Figure 1:**
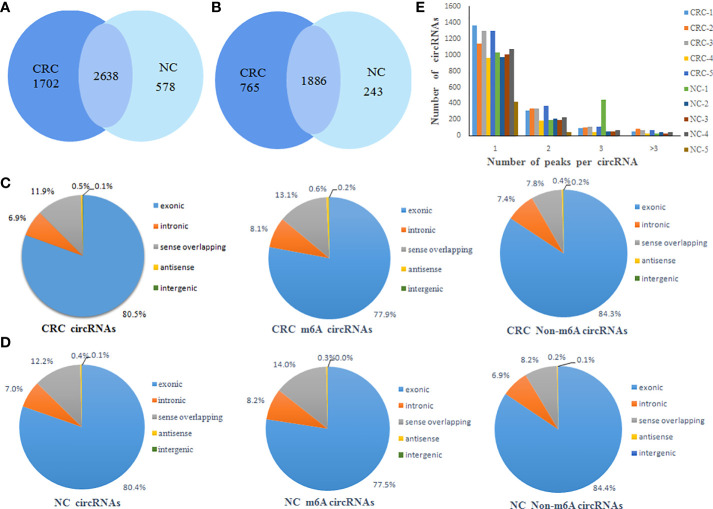
General characteristics of circRNA m^6^A modification patterns. **(A)** Venn diagram of the number of m^6^A peaks of circRNAs in CRC and NC groups; **(B)** Venn diagram of the number of m^6^A circRNAs in CRC and NC groups; **(C)** The distribution of the types of total circRNAs, m^6^A circRNAs and non-m^6^A circRNAs in CRC group; **(D)** The distribution of the types of total circRNAs, m^6^A circRNAs and non-m^6^A circRNAs in NC group; **(E)** Distribution of the number of circRNAs (y axis) plotted against the number of m^6^A peaks in each circRNA (x axis) for CRC and NC group.

A total of 4340 m^6^A peaks of circRNAs in CRC tissue and 3216 m^6^A peaks of circRNAs in NC tissues were detected. A total of 2561 m^6^A circRNAs in CRC tissues and 2129 m^6^A circRNAs in NC tissues were detected. There were 2638 overlapping m^6^A peaks of circRNAs and 1886 overlapping m^6^A circRNAs between the two groups. Compared to NC tissues, CRC tissues had 1702 unique m^6^A peaks of circRNAs and 765 m^6^A circRNAs. These results showed that there was a significant difference in the overall m^6^A modification pattern between CRC and NC tissues (**Figures 1A, B**).

CircRNAs can be classified into various types based on the type of sequences they contain. Therefore, we analyzed the distribution of the types of circRNAs represented in the total circRNAs, m^6^A circRNAs and non-m^6^A circRNAs groups from both the CRC and NC tissues. This analysis revealed that exonic circRNAs account for 80% of the total circRNAs, 77% of the m^6^A circRNAs, and 84% of the non-m^6^A circRNAs in CRC tissues (**Figure 1C**). Similarly, exonic circRNAs accounted for the highest proportion of transcripts in all three groups from NC tissues at approximately 80% (**Figure 1D**). This indicated that the exonic circRNAs were the most prevalent version of these circular RNAs, which was consistent with previous studies. In addition, it was found that most circRNAs had the unique m^6^A modified peak both CRC and NC tissues. A relatively small number of m^6^A modified genes contain two or more peaks (**Figure 1E**).

**Figure 2:**
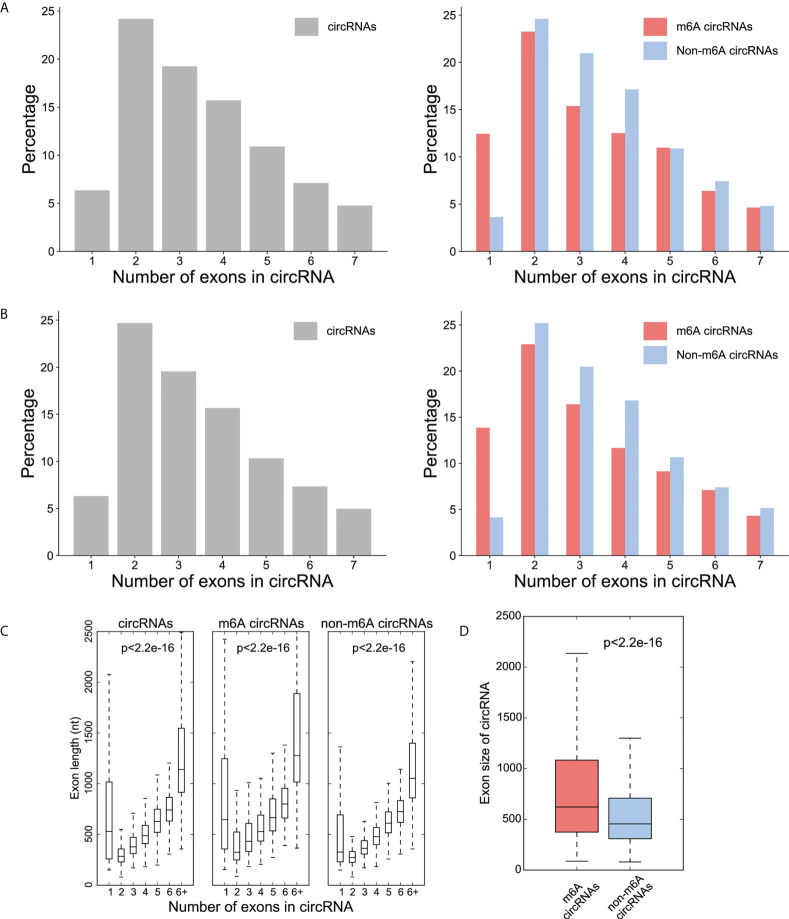
Distribution patterns for the exons in the circRNAs. **(A)** Percentage (y-axis) of exon number (x-axis) of circRNA (left), m^6^A -circRNA and non-m^6^A circRNA (right) in CRC group; **(B)** Percentage (y-axis) of exon number (x-axis) of circRNA (left), m^6^A -circRNA and non-m^6^A circRNA (right) in NC group; **(C)** The distribution of exon lengths (y axis) for each of the input circRNAs (left), m^6^A circRNAs (middle), and non-m^6^A circRNAs (right) were plotted against the number of exons (x axis) each circRNA spans. For all box plots, the lower edge of the box represents the first quartile and the upper edge represents the third quartile. The horizontal line inside the box represents the median and the whiskers identify the farthest data points within a 1.5 x interquartile range (IQR); **(D)** Comparison of exon size (nt) between m^6^A circRNAs and non-m^6^A circRNAs.

The majority of the total circRNAs from both CRC and NC groups contained two or three exons. The most common of the m^6^A circRNAs group and the non-m^6^A circRNAs group are circular RNA transcripts containing two exons (**Figures 2A, B**). The exon length analysis showed that circRNAs containing more than 6 exons were the longest with an average length of more than 1000bp, followed by single exon circRNAs (**Figure 2C**). In addition, the exon length of the m^6^A circRNAs was significantly longer than non-m^6^A circRNAs (**Figure 2D**).

### Distribution of differentially methylated m^6^A circRNAs

**Figure 3:**
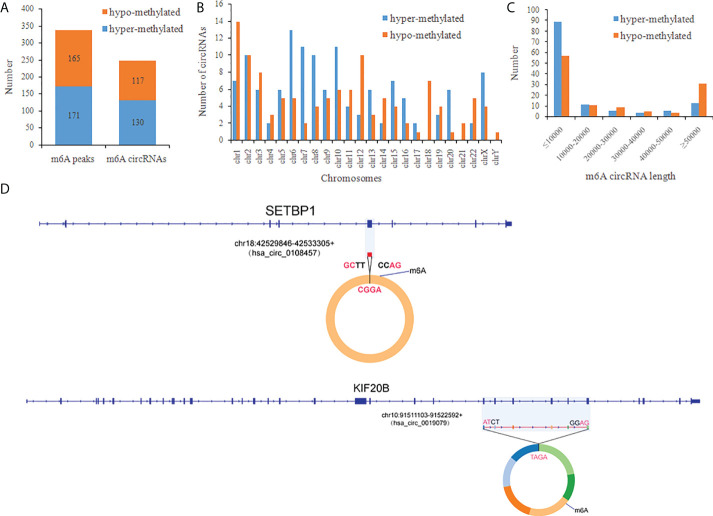
General characteristics of differentially methylated m^6^A circRNAs in CRC. **(A)** The number of differentially m^6^A peaks and differentially m^6^A circRNAs in CRC; **(B)** The chromosome origins for genes of these differentially methylated circRNAs; **(C)** The length of the differentially methylated circRNAs; **(D)** Schematic representation of the exons included in the hsa-circ-0019079 (circKIF20B) and has-circ-0108457 (circSETBP1) circularized transcripts.

There were 336 differential m^6^A peaks of circRNAs and 247 differential m^6^A circRNAs were found in CRC relative to the NC group. Meanwhile, There were 130 hyper-methylated circRNAs and 117 hypo-methylated circRNAs were found in CRC (**Figure 3A**). The top 10 circRNAs of m^6^A hypermethylated and hypomethylated with the highest fold-change values are shown in **Table 1**. The hyper-methylated circRNAs were predominantly located on chromosomes 6, 7 and 10, while the hypo-methylated circRNAs were predominantly located on chromosomes 1, 2, and 12 (**Figure 3B**). Moreover, The length of hyper-methylated and hypo-methylated m^6^A circRNAs is mainly enriched in ≤10000 bp (**Figure 3C**).

**Table 1:** The top 10 circRNAs of m^6^A hypermethylated or hypomethylated.

CircRNA		circBaseID		GeneSymbol	Catalog	Regulation	FC	P
chrX:130877124- 130928494-		novel		FIRRE	sense overlapping	Up	98.90	5.86E-09
chr10:4872867- 4950612+		hsa_circ_0093688		AKR1E2	sense overlapping	Up	76.80	7.42E-07
chr3:44882566- 44894286+		novel		KIF15	sense overlapping	Up	32.47	7.42E-08
chr13:60348323- 60413582		hsa_circ_0100755		DIAPH3	exonic	Up	14.40	2.81E-08
chr10:129901479- 129917583-		novel		MKI67	sense overlapping	Up	14.35	1.93E-07
chr20:6077549- 6100386-		novel		FERMT1	sense overlapping	Up	12.94	5.70E-07
chr2:99763865- 99767305+		novel		C2orf15	sense overlapping	Up	12.38	7.83E-09
chr10:63958042- 63983091-		novel		RTKN2	exonic	Up	9.85	2.83E-09
chr2:120885264- 120932576+		hsa_circ_0006834		EPB41L5	sense overlapping	Up	9.72	1.88E-07
Chr11: 6919324- 7018251-		novel		G012495	sense overlapping	Up	8.79	2.947E-07
chr8:56435840- 56439309+		hsa_circ_0136773		XKR4	sense overlapping	down	157.50	2.53E-09
chr16:15917112- 15932126-		novel		MYH11	exonic	down	128.11	5.14E-07
chr3:64795470- 64862129+		novel		MIR548A2	intronic	down	84.40	6.96E-09
chr12:10572963- 10588009-		novel		KLRC2	sense overlapping	down	74.10	7.05E-09
chr8:56435840- 56439309+		hsa_circ_0136773		XKR4	sense overlapping	Down	73.19	9.46E-09
chr3:67659879- 67688263-		novel		SUCLG2	sense overlapping	down	46.50	1.32E-07
chr18:31206826- 31263532+		hsa_circ_0108271		ASXL3	sense overlapping	down	43.50 1	68E-08
chr19:4511919- 4512215-		novel		XLOC_031263	Intronic	down	40.25	1.99E-07
chrX:32305646- 32366645-		hsa_circ_0140203		DMD	exonic	down	32.58	1.09E-07
chr14:35020920- 35024118-		hsa_circ_0007379		G024201	sense overlapping	down	27.68	2.90E-08

We confirmed the back-splicing of hsa_circ_0019079 of hyper-methylated circRNAs and has_circ_0108457 of hypo-methylated circRNAs using CIRI software. Has_circ_0019079 was located on chromosome 10 and spliced by 6 exons from KIF20B. Has_circ_0108457 was located on chromosome 18 and spliced by a single exon from SETBP1 (**Figure 3D**). Interestingly, both KIF20B and SETBP1 were involved in the development of CRC (33, 34).

### Differentially m^6^A modification circRNAs are involved in important biological pathways

**Figure 4:**
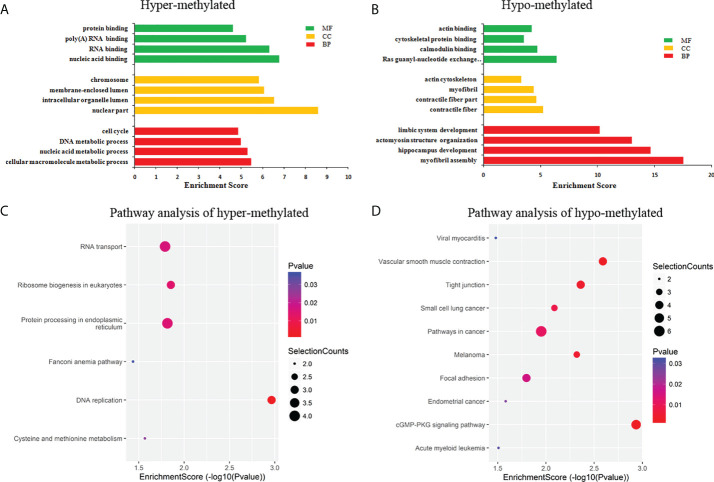
Pathway analysis of differentially m^6^A circRNAs. **(A)** GO analysis of hyper-methylated m^6^A circRNAs; **(B)** GO analysis of hypo-methylated m^6^A circRNAs; **(C)** KEGG analysis of hyper-methylated m^6^A circRNAs; **(D)** KEGG analysis of hypo-methylated m^6^A circRNAs.

GO and KEGG pathway analyses were performed to evaluate the biological significance of differentially m^6^A circRNAs. GO analysis shows that hyper-methylated circRNAs were mainly concentrated in cellular macromolecule metabolic processes, nuclear part and nucleic acid binding (**Figure 4A**). While hypo-methylated circRNAs were mainly enriched in myofibril assembly, contractile fiber and Ras guanyl-nucleotide exchange factor activity (**Figure 4B**). KEGG pathway analysis revealed that the hyper-methylated circRNAs were mainly involved in DNA replication, RNA transport, ribosome biogenesis in eukaryotes, and protein processing in the endoplasmic reticulum (**Figure 4C**). The hypo-methylated circRNAs were enriched in pathways of cancers, cGMP-PKG, and tight junction signaling pathways (**Figure 4D**).

### Differentially expressed circRNA profiles in CRC

**Figure 5:**
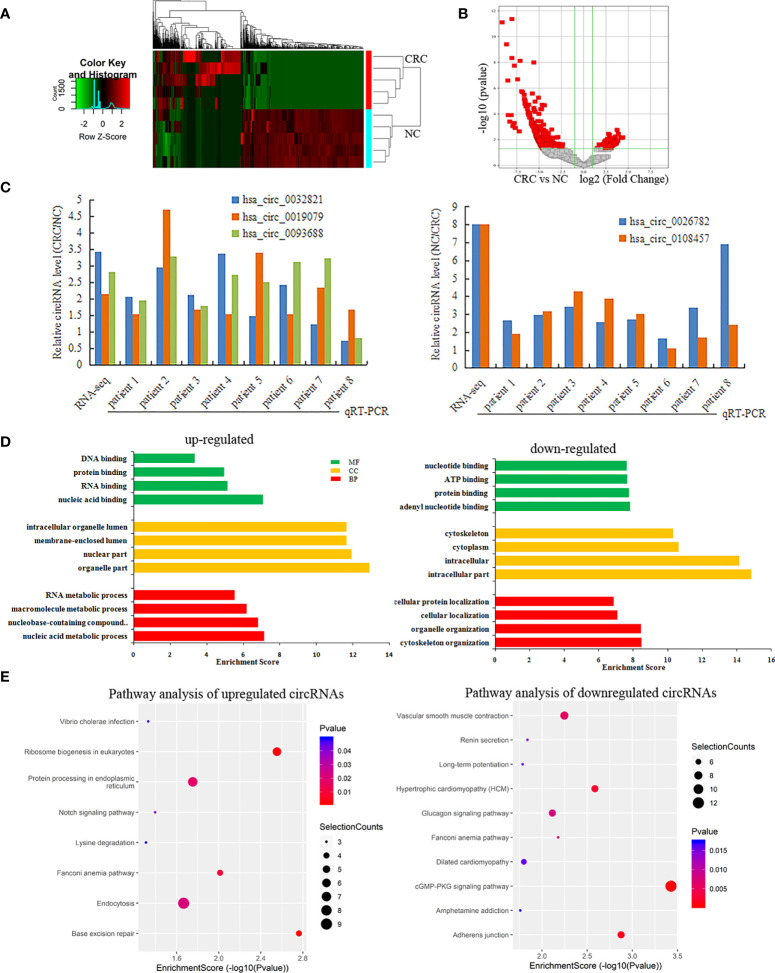
Differentially expressed circRNAs in CRC. **(A, B)** A heatmap hierarchical clustering and Volcano plot were used to visulize the differentially expressed circRNAs in each of the groups; **(C)** The RT-qPCR was used to detect the expression of 5 circRNAs in CRC and NC tissues; **(D)** GO enrichment analysis for Upregulated and Downregulated circRNAs; **(E)** KEGG pathway analysis for Upregulated and Downregulated circRNAs.

RNA-seq was used to detect differentially expressed circRNAs (fold-change ≥2 and p-value <0.05) in CRC and NC tissues, which showed that there was a significant difference in the expression of circRNAs (**Figure 5A**). The volcano plot shows that 877 circRNAs were differentially expressed, including 522 downregulated and 355 upregulated circRNAs in CRC (**Figure 5B**). The top 10 upregulated and downregulated genes are listed in **Table 2**.

**Table 2:** The top 10 upregulated or downregulated circRNAs.

CircRNA	circBaseID	strand	GeneSymbol	Catalog	Regulation	LogFC
chr9:4823548-	hsa_circ_0006134	+	RCL1	exonic	Up	4.404833228+
chr15:49528048-	hsa_circ_0035189	+	GALK2	exonic	Up	4.3049584734+
chr2:55209651-	hsa_circ_0001006	–	RTN4	exonic	Up	4.1755214834-
chr8:80963762-	hsa_circ_0137152	–	TPD52	exonic	Up	3.9080976828-
chr2:165548731-	hsa_circ_0056891	–	COBLL1	exonic	Up	3.84165561615-
chr19:41456345-	novel	+	G040696	intronic	Up	3.7541524082
chr13:96375496-	hsa_circ_0002473	+	DNAJC3	exonic	Up	3.7196377506+
chr2:61406116-	novel	+	AHSA2	exonic	Up	3.6461413632+
chr15:65471272-	hsa_circ_0004374	–	CLPX	exonic	Up	3.6465472542
chr10:124340382-	novel	+	DMBT1	exonic	Up	3.58124358613
chr4:151388825-	hsa_circ_0006867	–	LRBA	exonic	Down	9.14151412187-
chr6:151669846-	hsa_circ_0078299	+	AKAP12	exonic	Down	8.65151674887+
chr10:29801664-	novel	–	SVIL	exonic	Down	8.5029821101-
chr9:86506374-	novel	–	KIF27	sense overlapping	Down	8.4686514671-
chr15:65244791-	novel	–	ANKDD1A	sense overlapping	Down	8.2965244955-
chr18:51804073-	hsa_circ_0003428	+	POLI	exonic	Down	8.1251813781+
chr12:56094683-	hsa_circ_0026782	–	ITGA7	exonic	Down	8.0556094938-
chr18:42529846-	hsa_circ_0108457	+	SETBP1	exonic	Down	8.0542533305+
chrM:1692-1896+	novel	+	G087361	Intronic	Down	7.94
chr1:235530417-	novel	+	G007566	exonic	Down	7.92235564902+

Among these differentially expressed circRNAs, we used RT-qPCR to detect the expression of 5 circRNAs in 8 pairs of CRC and NC tissues. The results of RT-qPCR showed the same expression trend with the RNA-Seq results. Compared with NC tissue, CircRNAs (has-circ-0032821, has-circ-0019079, has-circ-0093688) were upregulated, while circRNAs (hsa_circ_0026782, hsa_circ_0108457) were downregulated in CRC (**Figure 5C**).

GO analysis revealed that the upregulated circRNAs were involved in the nucleic acid metabolic process, intracellular part and nucleotide binding. The downregulated circRNAs were involved in cytoskeleton organization, intracellular part and nucleotide binding (**Figure 5D**). KEGG pathway analysis showed that the upregulated circRNAs were closely related to base excision repair, ribosome biogenesis in eukaryotes, and protein processing in the endoplasmic reticulum, while the downregulated circRNAs were closely related to cGMP-PKG signaling pathway and adherens junctions (**Figure 5E**).

### Association between m^6^A methylation and circRNA expression

**Figure 6:**
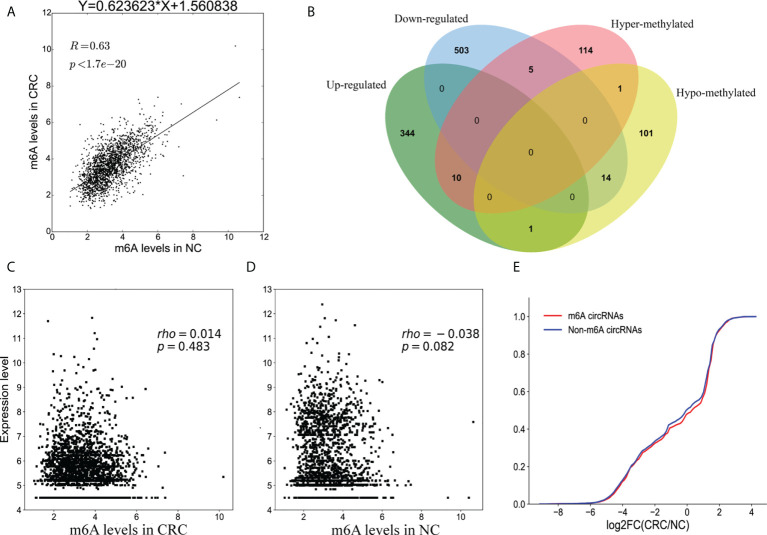
Association between m^6^A methylation and circRNAs expression. **(A)** Scatter plot showing the linear correlation between circRNAs expression and m^6^A methylation in CRC and NC group; **(B)** Venn diagram showing the relationship between m^6^A modifications and the expression of circRNAs; **(C)** Scatter plot of the correlation between m^6^A levels and circRNAs expression in CRC group; **(D)** Scatter plot of the correlation between m^6^A levels and circRNAs expression in NC group; **(E)** Cumulative distribution of circRNAs expression for m^6^A circRNAs (red) and non-m^6^A circRNAs (blue).

Methylation sequencing data showed that the m^6^A peaks in the circRNAs from CRC tissues was more than that of the NC tissues. Here, we analyzed the methylation correlation between the two groups. The results showed that the m^6^A level of circRNAs in the CRC was positively correlated with the m^6^A level of circRNAs in the NC group (Spearman’ s rho = 0.63, p <0.05) (**Figure 6A**).

Combining the results of the methylation sequencing and RNA sequencing, we identified 130 hypermethylated circRNAs, 10 of which were upregulated and 5 were downregulated. A total of 117 circRNAs were hypomethylated, 14 of which were downregulated and one was upregulated (**Figure 6B**). Interestingly, the methylation level of one of the circRNAs had both up-regulated and down-regulated m^6^A sites. To analyze the correlation between circRNA methylation and expression levels, we constructed a correlation graph using the fold enrichment of circRNA m^6^A methylation and expression values described by FPKM. The results indicate that there was a statistically significant positive correlation between methylation and expression of circRNAs in CRC and NC samples (**Figures 6C, D**).

To further analyze the m^6^A effects on circRNAs expression, we divided all of the circRNAs into m^6^A and non- m^6^A circRNAs groups. We then calculated the log two-fold change values for these circRNAs and generated a cumulative curve. There was no significant difference between m^6^A and non-m^6^A circRNAs (**Figure 6E**).

### Co-expression network of circRNA-miRNA-mRNA in CRC

**Figure 7:**
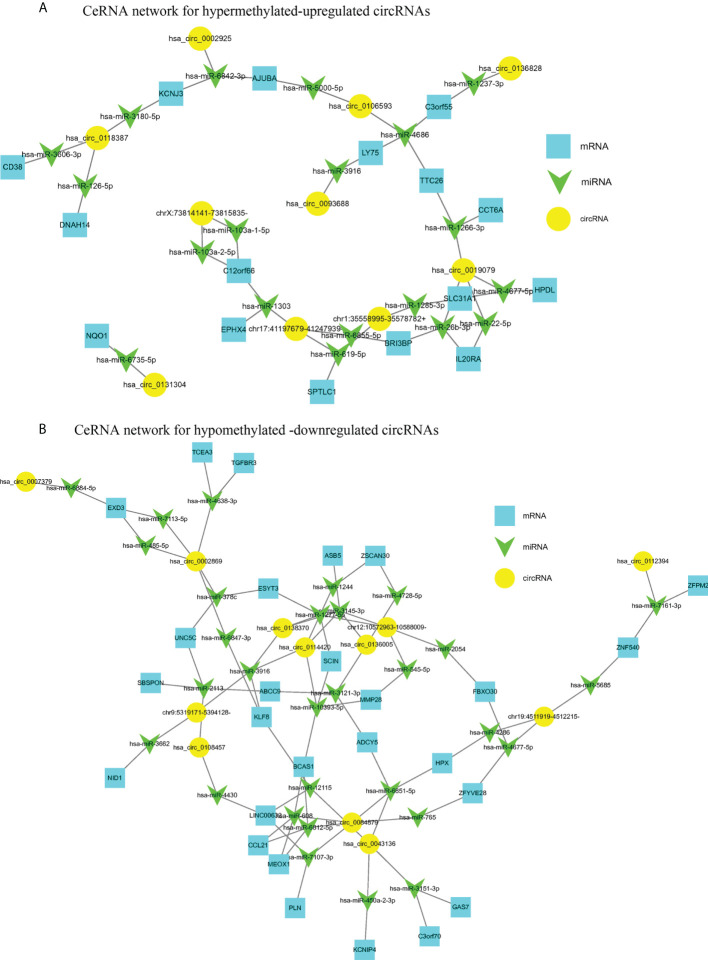
The circRNA-miRNA-mRNA networks in CRC. **(A)** CeRNA analysis for hyper-methylated upregulated circRNAs; **(B)** CeRNA analysis for hypo-methylated downregulated circRNAs.

CircRNAs are generally considered as sponges for miRNAs to fine-tune the miRNA-mRNA regulatory network. We selected 10 hyper-up circRNAs and 14 hypo-down circRNAs to establish the circRNA-miRNA-mRNA network. The 10 hyper-up and 14 hypo-down circRNAs are listed in **Table 3** and **Table 4**. In the ceRNA network, the 10 hyper-up circRNAs were shown to be associated with 19 miRNAs and regulate 16 mRNAs (**Figure 7A**). Similarly, 14 hypo-down circRNAs were associated with 30 miRNAs and regulated 27 mRNAs (**Figure 7B**). In addition, there was a positive correlation between circRNA and mRNA expression. when the expression of the circRNA was upregulated the expression of the target mRNA was upregulated (**Figure 7**). In these mRNAs, we found that 40 were associated with tumors and 28 of these were specifically associated with CRC through the GEPIA database. This suggests that m^6^A may regulate CRC related genes through the circRNA-miRNA-mRNA co-expression network (35–39).

**Table 3:** The 10 hypermethylated and upregulated genes.

CircRNA	circBaseID	Pattern	Gene Symbol	m^6^A change		mRNA change	
				FC	P	LogFC	P
chr1:4872867- 4950612+	hsa_circ_0093688	Hyper-up	AKR1E2	76.80	7.42E-07	2.81	8.77E-03
chr10:91511103- 91522592+	hsa_circ_0019079	Hyper-up	KIF20B	7.46	4.53E-08	2.15	2.50E-02
chr17:30320261- 30323896+	hsa_circ_0106593	Hyper-up	SUZ12	5.49	5.38E-06	2.64	4.00E-02
chr17:41197679- 41247939-	novel	Hyper-up	BRCA1	5.29	4.91E-07	2.42	4.62E-02
chr2:190717381- 190719854+	hsa_circ_0118387	Hyper-up	PMS1	3.53	1.96E-07	2.42	4.62E-02
chrX:73814141- 73815835-	novel	Hyper-up	RLIM	3.21	9.58E-08	2.80	2.73E-02
chr9:115336337- 115337531+	hsa_circ_0002925	Hyper-up	KIAA1958	2.74	2.87E-07	3.07	2.50E-02
chr6:167435897- 167453827+	hsa_circ_0131304	Hyper-up	FGFR1OP	2.67	2.77E-09	2.99	3.34E-02
chr1:35558995- 35578782+	novel	Hyper-up	ZMYM1	2.64	9.67E-07	2.64	3.40E-02
chr8:61707545- 61714152+	hsa_circ_0136828	Hyper-up	CHD7	2.43	1.74E-07	2.68	3.87E-02

**Table 4:** The 14 hypomethylated and downregulated genes.

CircRNA	circBaseID	Pattern	Gene Symbol	m^6^A change		mRNA change	
				FC	P	LogFC	P
chr12:10572963- 10588009-	novel	Hypo-down	KLRC2	74.10	7.05E-09	4.90	1.82E-02
chr19:4511919- 4512215-	novel	Hypo-down	XLOC_031263	40.25	1.99E-07	4.91	1.45E-02
chr14:35020920- 35024118-	hsa_circ_0007379	Hypo-down	G024201	27.68	2.90E-08	3.22	4.55E-02
chr18:42529846- 42533305+	hsa_circ_0108457	Hypo-down	SETBP1	9.20	9.33E-07	8.05	4.61E-09
chr17:34105892- 34106327-	hsa_circ_0043136	Hypo-down	MMP28	8.89	1.25E-08	5.33	5.07E-03
chr1:240328989- 240351562+	hsa_circ_0003470	Hypo-down	FMN2	8.63	3.16E-07	7.78	5.15E-04
chr3:71733723- 71748859-	hsa_circ_0124568	Hypo-down	EIF4E3	4.60	8.41E-09	4.60	3.13E-02
chr1:201681945- 201687883+	hsa_circ_0002869	Hypo-down	NAV1	4.30	4.71E-07	4.56	4.54E-02
chr1:232596633- 232607274-	hsa_circ_0112394	Hypo-down	SIPA1L2	3.17	8.70E-10	4.86	2.25E-02
chr1:91382197- 91406866-	hsa_circ_0114420	Hypo-down	ZNF644	2.21	3.58E-08	4.63	4.34E-02
chr8:16955967- 16977910+	hsa_circ_0136005	Hypo-down	MICU3	4.70	6.23E-08	5.20	5.84E-03
chr8:92998352- 93029591-	hsa_circ_0084879	Hypo-down	RUNX1T1	7.23	1.03E-07	5.05	1.02E-02
chr9:5319171- 5394128-	novel	Hypo-down	RLN1	3.87	6.31E-07	4.63	4.34E-02
chr9:18336330- 18382644+	hsa_circ_0138370	Hypo-down	GSE61474_TCONS_00326176	7.11	3.96E-08	4.58	1.70E-02

### Relationship between circRNAs with coding potential and methylation

**Figure 8:**
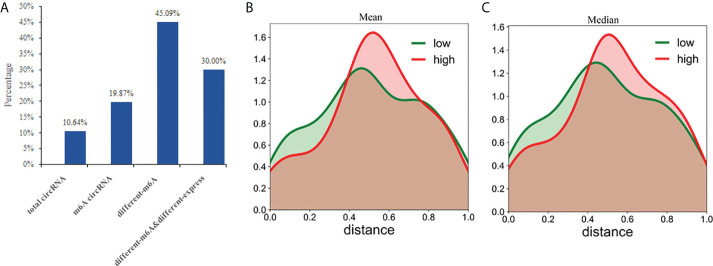
CircRNAs with coding potential predominantly exhibit N^6^-adenosine methylation. **(A)** The percentages of circRNAs with protein-coding potential in total circRNAs, m^6^A circRNAs, differentially m^6^A circRNAs, and differentially m^6^A& differentially expressed circRNAs were compared; The distance between the methylation peak and the ORFs for each of the circRNAs was calculated and then the mean **(B)** or median **(C)** value of their coding potential scores were used to stratify the circRNAs into low and high probability groups which were then used to create the density function diagram.

Previously, it was thought that circRNAs could not encode any functional products, but recent studies have shown that circRNAs also possess some coding potential with a handful even encoding putative proteins (24–26). In this study, we analyzed the protein-coding potential of circRNAs using LGC software (32). The results showed that 850 of the 7990 (10.64%) circRNAs have a certain protein coding potential. In the 2894 m^6^A circRNAs, 575 had protein-coding potential, accounting for 19.87%. In the 224 differentially methylated circRNAs, 101 have protein-coding potential, accounting for 45.09%. Of the 30 circRNAs exhibiting both differential methylation and expression, nine were shown to have protein-coding potential, accounting for 30.00% (**Figure 8A**). Compared with the total circRNA, there are significantly more circRNAs with protein coding potential in the m^6^A group. As a result, m^6^A may increase the coding potential of circRNAs.

CircRNAs can be translated with an internal ribosome entry site (IRES) (40, 41). And m^6^A can act as an IRES to drive circRNAs translation in a cap-independent manner with even a single m^6^A site being sufficient to initiate translation (42). Some non-coding RNAs are reported to have coding potential from 5′ open reading frames (ORFs) (43). This suggests that the ORFs commonly found in circRNAs may be translated by internal m^6^A sites. Therefore, we evaluated whether the distance between the m^6^A peak and ORFs could act as a deciding factor for their translation. Based on the mean or median value of their coding potential score, the circRNAs were divided into low and high groups and the density function diagram was drawn (**Figures 8B, C**). The results showed that the distance of high coding potential score was farther from IRES.

## Discussion

In this study, we managed to profile circRNAs that were differentially methylated and expressed in CRC and NC tissues. These results showed that there was a significant difference in m^6^A methylation and expression of circRNAs between the CRC and NC tissues. There are several reported circRNAs arising from the exonic regions that contribute to cancer progression in CRC (44, 45). Our study revealed that exonic circRNAs account for 80% of the total circRNAs, 77% of the m^6^A circRNAs, and 84% of the non-m^6^A circRNAs in CRC tissues. The m^6^A methylation levels in the CRC group were significantly higher than NC group. There was a significant positive correlation between methylation and expression levels. In addition, m^6^A modification can affect the expression of circRNAs altering their fine-tuning of the miRNA-mRNA regulatory axis, and thus affecting the expression of tumor-related target mRNAs. We found that methylation affected the coding potential of the circRNAs but this effect was not related to the distance between the m^6^A peak and ORFs.

CircRNAs occur in the nucleus, and most circRNAs containing introns are confined to the nucleus but most of the exon circRNAs are localized to the cytoplasm (16). The m^6^A binding protein YTHDC1 regulates the export of methylated mRNA and can also regulate the transfer of m^6^A circRNAs from the nucleus to the cytoplasm (46). M^6^A modified circNSUN2 is exported to the cytoplasm by YTHDC1 and functions to improve the stability of HMGA2 mRNA and promote liver metastasis in CRC (44). Our results show that the methylation level of circRNAs in CRC tissues is significantly increased and that this methylation is mainly concentrated in circRNAs composed of long exons. some circRNAs arising from the exonic regions contribute to the development and progression of this cancer (44, 45). The exon circRNAs with methylation may be worthy of further study in colorectal cancer.

Circ_0032821 was significantly upregulated in human GC tumors and cells. its’ expression induced cell proliferation, EMT, migration, invasion and autophagy inhibition in human GC cells through activating MEK1/ERK1/2 signaling pathway (46). Circ_0032821 was also highly expressed in OXA-resistant GC cells and contributes to oxaliplatin (OXA) resistance of gastric cancer cells by regulating SOX9 *via* miR-515-5p (47). Hsa_circ_0026782 (circITGA7) and its linear host gene ITGA7 are both significantly downregulated in CRC tissues and cell lines. These decreased expression levels correlated with CRC progression. They play a suppressor in CRC. CircITGA7 inhibits the proliferation and metastasis of CRC cells by suppressing the Ras signalling pathway and promoting the transcription of ITGA7 (22). In another study found that circITGA7 sponges miR-3187-3p to upregulate ASXL1, suppressing colorectal cancer proliferation (48). ITGA7 also plays an important tumorigenic function and acts as a suppress gene in breast cancer (49). In this study, we found that circ_0032821 were upregulated and hsa_circ_0026782 were downregulated in CRC. The expression trend of those circRNAs is consistent. In addition, We verified that hsa_circ_0019079 (circKIF20B) were upregulated and has_circ_0108457 (circSETBP1) were downregulated in CRC tissues. There is no research on hsa-circ-0019079 and has-circ-0108457. However, both of KIF20B and SETBP1 are involved in the development of CRC (33, 34). Their role in colorectal cancer is worthy of further study.

By cross analyzing the m^6^A-Seq and RNA-seq data, we identified 30 circRNAs with differential methylation and expression. 33.3% (10/30) of the host genes (KIF20B, BRCA1, NAV1, SIPA1L2, FMN2, MMP28, SETBP1, EIF4E3, MICU3, and RUNX1T1) were associated with CRC. The expressions of NAV1, SIPA1L2, FMN2, MMP28, SETBP1, EIF4E3, MICU3, and RUNX1T1 have shown to be decreased in CRC samples and reduced expression levels of SIPA1L2, MMP28, and EIF4E3 are associated with lower overall survival rates in CRC patients. KIF20B is upregulated in CRC promoting the migration and invasion of CRC (33). BRCA1 is associated with CRC and breast cancer (50, 51). MMP28 is involved in the occurrence and metastasis of gastric cancer and CRC (36, 52). Therefore, those circRNAs with differential methylation and expression patterns deserve further study, which may help to clarify the molecular function underlying the occurrence and development of CRC.

When we established a circRNA-miRNA-mRNA network by integrating matched circRNAs, miRNAs, and mRNAs expression profiles we were able to demonstrate a positive correlation between the expression of circRNAs and mRNA. We found that 30 differentially methylated circRNAs were associated with 40 tumor-related mRNAs and 28 of these were associated with CRC. For example, circRNAs hsa_circ_0106593 and hsa_circ_000292 serve as sponges for hsa-miR-5000-5p and hsa-miR-6842-3p, respectively, regulating the expression of the target gene AJUBA. AJUBA promotes the growth of CRC by inhibiting apoptosis (30). circRNA hsa_circ_008487 binds to hsa-miR-608 and hsa-miR-6812-5p, which bind to CCL21, which further inhibits the migration and invasion of CRC cells (31). circRNAs hsa_circ_0019079 and chr1:35558995-35578782+ positively regulate the expression of SLC31A1, hsa_circ_0114420, and chr12:10572963-10588009- which can positively regulate the expression of MMP28. The expression of ZFPM2 decreases with decreasing hsa_circ_0112394 expression and SLC31A1, MMP28, and ZFPM2 are associated with the occurrence, progression, and prognosis of CRC (35, 36, 39). M^6^A is a post-transcriptional modification which regulates circRNAs expression. We speculate that m^6^A could be involved in the occurrence and development of CRC by affecting the circRNA-miRNA-mRNA co-expression networks. This may provide a theory for the mechanism underlying circRNA activity in CRC. Furthermore, regulating m^6^A modifications may be a future strategy for the treatment of CRC.

CircRNAs are defined as non-coding RNAs. however, it has recently been shown that some circRNAs actually encode proteins, and that these proteins are involved in the occurrence, development and drug resistance of many tumors (22–24). The 5’ and 3’ untranslated regions (UTRs) are essential elements for canonical cap-dependent translation in eukaryotic cells. Due to the lack of the 5 ‘cap and 3’ end, the translation of circRNA can only be initiated in a cap-independent manner. IRES- and m^6^A- mediated cap-independent translation initiation are accepted as important mechanisms for circRNA translation (40–42). IRESs are sequences located in the 5’ UTR of mRNAs that directly recruit ribosomes to initiate translation (53). M^6^A in the 5’ UTR can directly bind to eukaryotic initiation factor 3 (eIF3) prompting translation (54). M^6^A driven translation is an alternative mechanism often employed under stress conditions (55). Interfering with the m^6^A modification level in RNAs can influence translation efficiency (42, 56) and a single m^6^A site is sufficient to initiate circRNA translation *via* eIF4G2 and m^6^A reader YTHDF3 (42). In this study, we predicted the protein-coding potential of all the circRNAs and found that methylation increases the coding potential of circRNA transcripts. There were significantly more circRNAs with protein-coding potential in the m^6^A circRNA group and the proportion (45.09%) of potentially coding transcripts was highest in the differentially methylated circRNAs. This may be because these short RNA elements containing m^6^A sites have IRES-like activity initiating the translation of the circRNAs (42). We further analyzed if the distance between the methylation site and ORFs may predict greater protein coding potential. However, further analysis of the density function diagram indicated that methylation affected the coding potential of the circRNAs, but this effect was not related to the distance between the methylation peak and the ORFs.

Our study found that there were significant differences in the m^6^A methylation patterns of circRNAs between CRC and NC tissues and that the level of m^6^A methylation could affect the expression level and increase the coding potential of circRNAs. Bioinformatic analyses showed that the host genes of differentially methylated circRNAs were associated with tumor development-related pathways. In addition, we showed that m^6^A methylation may affect circRNA-miRNA-mRNA co-expression in CRC and further affect the regulation of cancer-related target genes. However, the differentially methylated circRNAs contribute to the occurrence and development of CRC needs further study. In our follow-up research, I will target the circRNAs selected in this experiment, such as hsa_circ_0093688, hsa_circ_0019079, hsa_circ_0108457, hsa_circ_0043136. Methyltransferase METTL3/METTL14, demethylase FTO/ALKBH5, methylation reader YTHDF/IGF2BP2 will be used to verify the methylation expression level of circRNAs, and functional exploration will be carried out based on KEGG and GO results. The network map of circRNA-miRNA-mRNA was used to find the regulated target genes for mechanism research.

## Conclusions

This study found that there were significant differences in the m^6^A methylation patterns of circRNAs between CRC and NC tissues. M^6^A methylation may affect circRNA-miRNA-mRNA co-expression in CRC and further affect the regulation of cancer-related target genes.

## Data availability statement

The datasets presented in this study can be found in online repositories. The names of the repository/repositories and accession number(s) can be found below: https://www.ncbi.nlm.nih.gov/geo/query/acc.cgi?acc=GSE190388.

## Ethics statement

The studies involving human participants were reviewed and approved by The First Affiliated Hospital of Chengdu Medical College. The patients/participants provided their written informed consent to participate in this study. Written informed consent was obtained from the individual(s) for the publication of any potentially identifiable images or data included in this article.

## Author contributions

YZ and FH were involved in the conception and design of the study. QG and GXJ acquired the data and drafted the manuscript. QG collected the samples and conducted data analysis. YZ critically revised the manuscript for intellectual content, approved the final version. All authors read and approved the final manuscript.

## Funding

The present study was supported by the scientific research project of Sichuan Provincial Health Commission (grant No. 19PJ190), Mianyang Central Hospital hospital-level project (grant No. 2021YJRC-002).

## Conflict of interest

The authors declare that the research was conducted in the absence of any commercial or financial relationships that could be construed as a potential conflict of interest.

## Publisher’s note

All claims expressed in this article are solely those of the authors and do not necessarily represent those of their affiliated organizations, or those of the publisher, the editors and the reviewers. Any product that may be evaluated in this article, or claim that may be made by its manufacturer, is not guaranteed or endorsed by the publisher.
